# A Phenotypic and Genotypic Evaluation of Developmental Toxicity of Polyhexamethylene Guanidine Phosphate Using Zebrafish Embryo/Larvae

**DOI:** 10.3390/toxics8020033

**Published:** 2020-05-02

**Authors:** Jeongah Song, Kojo Eghan, Sangwoo Lee, Jong-Su Park, Seokjoo Yoon, Wittaya Pimtong, Woo-Keun Kim

**Affiliations:** 1 Jeonbuk Department of Inhalation Research, Korea Institute of Toxicology, Jeongeup 56212, Korea; jasong@kitox.re.kr; 2 Department of Predictive Toxicology, Korea Institute of Toxicology, Daejeon 34114, Korea; keghan@kitox.re.kr (K.E.); sangwoo.lee@kitox.re.kr (S.L.); jongsu.park@kitox.re.kr (J.-S.P.); sjyoon@kitox.re.kr (S.Y.); 3 Human and Environmental Toxicology, University of Science and Technology, Daejeon 34113, Korea; 4 Nano Environmental and Health Safety Research Team, National Nanotechnology Center, National Science and Technology Development Agency, Pathum Thani 12120, Thailand; wittaya.pimtong@nanotec.or.th

**Keywords:** polyhexamethylene guanidine-phosphate, RNA sequencing, inflammation, embryotoxicity, pulmonary illness

## Abstract

Polyhexamethylene guanidine-phosphate (PHMG-P), a guanidine-based cationic antimicrobial polymer, is an effective antimicrobial biocide, potent even at low concentrations. Due to its resilient bactericidal properties, it has been used extensively in consumer products. It was safely used until its use in humidifiers led to a catastrophic event in South Korea. Epidemiological studies have linked the use of PHMG-P as a humidifier disinfectant to pulmonary fibrosis. However, little is known about its harmful impacts other than pulmonary fibrosis. Thus, we applied a zebrafish embryo/larvae model to evaluate developmental and cardiotoxic effects and transcriptome changes using RNA-sequencing. Zebrafish embryos were exposed to 0.1, 0.2, 0.3, 0.4, 0.5, 1, and 2 mg/L of PHMG-P from 3 h to 96 h post fertilization. 2 mg/L of PHMG-P resulted in total mortality and an LC_50_ value at 96 h was determined at 1.18 mg/L. Significant developmental changes were not observed but the heart rate of zebrafish larvae was significantly altered. In transcriptome analysis, immune and inflammatory responses were significantly affected similarly to those in epidemiological studies. Our qPCR analysis (Itgb1b, TNC, Arg1, Arg2, IL-1β, Serpine-1, and Ptgs2b) also confirmed this following a 96 h exposure to 0.4 mg/L of PHMG-P. Based on our results, PHMG-P might induce lethal and cardiotoxic effects in zebrafish, and crucial transcriptome changes were linked to immune and inflammatory response.

## 1. Introduction

A number of substituted guanidines have been identified as having antimicrobial properties, and they have been explored as therapeutics and disinfectants for industrial and household items and everyday use [1]. In 2001, polyhexamethylene guanidine-phosphate (PHMG-P) was synthesized with the aim of producing an effective, low atomic weighted, and cationic bactericide with low toxicity and irritation [2]. It was revealed to have very strong bactericidal properties [3]. Thus, it has been widely used in various consumer products, such as: detergents, humidifiers, air conditioners, heaters, and tattoo pigments [4]. However, this chemical induced severe toxic effects in some cases. In South Korea, PHMG-P, when used as a humidifier disinfectant, has caused catastrophic casualties. Following the Korea Centers for Disease Control and Prevention (KCDC) reports, it was revealed to have caused lung fibrosis causing casualties [5]. Nevertheless, PHMG-related materials are still considered in industrial areas due to its strong bactericidal properties. It has been reported previously that PHMG-P had mild cumulative toxic effects in mice that were exposed via the intragastric route, as well as low toxicity when rats were acutely exposed via the dermal route [6,7]. It was also reported that an intraperitoneal injection of 50 mg/kg per day to rats caused acute inflammation resulting in hepatitis [8].

Studies of exposure to PHMG-P via the respiratory tract have been carried out more extensively after the unfortunate incident in South Korea. Song et al. [9] showed in an acute study of mice that intratracheally exposing their lungs to PHMG-P led to lung fibrosis caused by thymic atrophy, which stimulated lymphoid cell depletion. Experimental intratracheal exposure of PHMG-P to male rats also resulted in increases in inflammatory cytokines and fibronectin mRNA, which culminated in pulmonary fibrosis [10]. In another study, an upregulation of fibronectin-1, a gene related to lung fibrosis, was observed in mice in an inhalation chamber with PHMG-P over 10 weeks. Furthermore, the downregulation of the caveolin-1 gene was found, which implies an aggravated pulmonary disease. It can lead to lung carcinogenesis attributable to the loss of tumor suppression activity [11]. Despite these studies in the past few years, the focus has mainly been on lung fibrosis and inflammation. Other adverse effects are not yet fully understood.

*Danio rerio*, the zebrafish, is a fast-growing and incredibly cost-effective model for examining vertebrate development studies. It has a rapid organogenesis, with complete organ development at about 120 h after fertilization. The zebrafish has high genomic and molecular similarities to humans and other vertebrates, and zebrafish gills are similar to mammalian lungs [12]. Hence, it is highly applicable to assessing the effects of chemicals in humans [13].

As an omics tool, RNA sequencing can be applied to deepen our understanding of normal physiological and pathological processes and to discover biomarkers that can be used for safety assessments, including the identification or prediction of toxicities [14]. The changes in expression of a wide range of genes upon exposure to toxicants can be determined by RNA sequencing and utilized to elucidate the mechanisms of toxicants [11,15].

In this study, our aim is to improve our understanding and help identify toxic effects induced by PHMG-P. To this end, we exposed zebrafish embryos/larvae to PHMG-P and conducted a developmental- and cardio-toxicity test and analyzed transcriptome changes.

## 2. Materials and Methods

### 2.1. Test Chemicals and Solution

PHMG-P (CAS No: 89697-78-9), purchased from BOC Sciences (Shirley, NY, USA) with a stock concentration of 256 mg/mL, was diluted to the required concentrations using E3 medium. E3 medium was prepared with 5 mM NaCl, 0.17 mM KCL, 0.33 mM CaCl_2_, and 0.33 mM MgSO_4_ as outlined in *Zebrafish: A Practical Approach* [16]. Chemicals used for the preparation of E3 medium were of analytical grade and obtained from Sigma-Aldrich (St. Louis, MO, USA).

### 2.2. Zebrafish Embryos

Wild type AB zebrafish (*Danio rerio*) were maintained under a 12:12 h light:dark cycle at a temperature of 28 °C, with routine checks of the water quality (temperature, dissolved oxygen, pH, and conductivity), at the Department of Predictive Toxicology, Korea Institute of Toxicology, Daejeon. Brine shrimp (*Artemia nauplii*) purchased from Inve Aquaculture, Inc. (Salt Lake City, UT, USA) were cultured as feed for the zebrafish. Zebrafish were fed freshly hatched brine shrimp twice daily. Fully fertilized and healthy embryos collected from naturally spawned fish were incubated in E3 medium at 28.5 °C prior to chemical exposure.

### 2.3. Embryonic Toxicity

Embryos at ~3 h post fertilization (hpf) were inspected using an Olympus 8ZX7 light microscope (Olympus Corporation, Waltham, MA, USA)., randomly selected, and kept in 100 mm diameter petri dishes containing 8 mL of E3 medium. Four replicates, which each had ten embryos, were prepared for the control and exposed group. Test concentrations (0.1, 0.2, 0.3, 0.4, 0.5, 1.0, and 2.0 mg/L) were determined based on preliminary exposure test results. Ten healthy embryos per well were carefully transferred into 12 well plates containing 2 mL of test solution. E3 medium for both the control and the exposed group was renewed at 24 h intervals. The zebrafish embryos/larvae in the well plates were placed in an incubator (JSR incubator JSCC-250CP (Gongju, Chungchungnam-Do, Korea)) at 28.5 °C with a 12:12 h light:dark photoperiod.

All exposure experiments were conducted in accordance with protocols approved by the Institutional Animal Care and Use Committee (IACUC) of the Korea Institute of Toxicology. Approval date 29 July 2019 (Protocol No. KIT-1907-0263).

### 2.4. Embryo/Larva Imaging

The accompanying images are of living embryos/larvae at 24, 48, 72, and 96 hpf. Individual and group photographs were taken with a Leica M205FA fluorescent microscope mounted with a Leica DFC 7000T camera module (Leica Camera AG, Wetzlar, Germany).

At 24 hpf, group photos were taken at 20× magnification in E3 medium. Larvae were captured at 32× magnification after 24 hpf. The larvae were anesthetized using tricaine methane sulfonate (0.004% *w*/*v*) and transferred to a glass slide containing methylcellulose to enable correct alignment. Coagulation of embryos, somite formation, non-detachment of tail, and absence of active heartbeat were all observed to assess the survival and developmental progress. Morphological parameters including bent spines, head malformation, and pericardial edema were also checked.

### 2.5. Heart Rate Count

Using a Leica M205FA fluorescent microscope, the heart rate of the embryos/larvae was evaluated and expressed in beats per minute (bpm). A counter and a timer were used to count the heartbeats of zebrafish embryos/larvae placed in the lateral position. Five organisms per group were evaluated at each time point. To prevent abrupt movement and wiggling during counting, the zebrafish embryos/larvae were anaesthetized with tricaine methane sulfonate in embryo media and fixed on a microscope glass slide containing 3% methylcellulose. Using a tricaine methane sulfonate concentration of 0.004% (*w*/*v*) has been shown not to affect heart rates in zebrafish embryos/larvae [17].

### 2.6. RNA Sequencing

For the RNA sequencing, 25 embryos were exposed to 0.4 mg/L of PHMG-P, corresponding to the lethal concentration LC_15_ for up to 96 h. This was done in 6 well plates containing 3 mL of control or test solution per well. The medium was replaced every 24 h during incubation. At 96 h exposure, 25 zebrafish larvae were collected independently from each of the three replicates for both the control and the exposed groups, then kept in RNAlater stabilization solution (Qiagen, Hilden, Germany) for further RNA extraction. Total RNA was extracted using the RNeasy mini kit (Qiagen, Hilden, Germany). rRNA band integrity was assessed using an Agilent RNA 6000 Nano kit (Agilent Technologies, CA, USA). Samples with an RNA Integrity Number (RIN) greater than 7 were utilized to construct the RNA library. Prior to cDNA library construction, 1 µg of total RNA and magnetic beads with oligo (dT) was used to enrich the poly (A) mRNA. Then, the refined mRNA was agitated into short fragments, and the double-stranded cDNA was instantaneously synthesized. The cDNA was subjected to end-repair poly (A) addition and linked with sequencing adapters using the TruSeq RNA Sample Prep Kit (Illumina, Ca, USA). The suitable fragments, purified via a Blue Pippin 2% agarose gel cassette (Sage Science, MA, USA), were selected as templates for PCR amplification. The final libraries were quantified using a KAPA library quantification kit (KAPA Biosystems, South Africa), and the quality of the library was assessed using an Agilent 2100 bioanalyzer (Agilent Technologies, CA, USA). These fragments comprised between 350 and 450 base pairs. Subsequently, the library was sequenced using an Illumina HiSeq2500 sequencing platform (Illumina, CA, USA). Low-quality reads were sieved according to the following benchmarks: reads contained more than 10% skipped bases, reads that contained more than 40% bases whose quality scores were less than 20, and reads with average quality scores of less than 20. The filtered reads were mapped to the zebrafish reference genome (Ensembl version 86, (EMBL-EBI, Hinxton, Cambridge, UK, 2019)) using the aligner STAR v.2.3.0e (STAR, Cold Spring Harbor, NY, USA, 2014).

### 2.7. GO Analysis

We measured gene expression levels using Cufflinks v2.1.1 (Trapnell Lab, Berkeley, CA, USA, 2013) [18] with the gene annotation database from Ensembl version 86. To improve the accuracy, we applied multi-read correction and frag-bias correction. The abundance of gene transcripts was measured in fragments per kilobase of transcript per million fragments mapped (FPKM). For differential expression analysis, gene level count data were generated using the HTSeq-count v0.5.4p3 tool (EMBL, Heidelberg, Germany, 2013) [19]. Using calculated read count data, differently expressed genes (DEGs) were identified using the Tag Count Comparison R package [20]. Based on the DEGs list, gene ontology (GO) enrichment was derived. The ontology and annotation files for GO enrichment analysis were downloaded from the gene ontology website (http://www.geneontology.org/). *p*-values <0.001 were considered statistically significant [21].

### 2.8. Gene Expression Measurement

To validate the RNA sequencing data, real-time PCR was performed with inflammation-related genes reported previously [11,16]. For real-time PCR assay, an extra 25 eggs at ~3 h after fertilization were exposed to 0.4 µg/mL PHMG-P, via the same method as used for the RNA sequencing. At 96 h exposure, 25 zebrafish larvae were collected and then kept in RNAlater stabilization solution (Qiagen, Hilden, Germany). Total RNA was extracted using RNeasy Mini kit (Qiagen, Hilden, Germany), in accordance with the manufacturer’s protocol. RNA yield and purity were determined using a Nanodrop ND-1000 spectrophotometer (Nanodrop Technologies, Wilmington, ED, USA). RNA samples with A_260/280_ and A_260/230_ >1.8 were used. One microgram of total RNA was transcribed using GoScript^TM^ reverse transcription System (Promega, Madison, WI, USA) according to the manufacturer’s instructions. PCR primers were designed using Genscript Real-time PCR Primer Design (https://www.genscript.com/tools/real-time-pcr-tagman-primer-design-tool). List of primers used, including their GenBank IDs are listed in Table S1. The specificity of the primers was checked using the melting curve and agarose gel electrophoresis. mRNA levels were quantified using SYBR Green on the ABI StepOne Plus instrument (Applied Biosystems, Foster City, CA, USA). PCR reactions were performed with a master mix containing 10 μL of SYBR Green PCR Master Mix (Applied Biosystems, Woolston, Warrington, UK), 0.5 μL of each primer (10 pmol), 3 μL of cDNA, and distilled water to a final volume of 20 μL. The PCR conditions were 95 °C for 10 min followed by 40 cycles of 15 sec at 95 °C and 1 min at 60 °C. The relative expression levels were calculated using the ΔΔ*C*t method [22] compared with the control and were presented as fold change. Ribosomal protein P0 (RPP0) was used as a reference gene. All reactions were carried out in three biological replicates and two technical replicates.

### 2.9. Statistical Analysis

For the normality and homogeneity of each variance, the Shapiro–Wilk test and the Levene’s test were used, respectively. To determine significant differences between the control and the PHMG-P exposure groups, one-way analysis of variance (ANOVA) with Dunnett’s test was carried out using SPSS 12 for Windows^®^ (SPSS, Chicago, IL, USA, 2005). TOXSTAT software version 3.5 (West Inc., Cheyenne, WY, USA, 1996) was used for calculating median lethal concentration (LC_50_) and 95% confidence interval (CI). Statistical significance was determined at *p* = 0.05.

## 3. Results

### 3.1. Mortality and Developmental Toxicity

**Figure 1:**
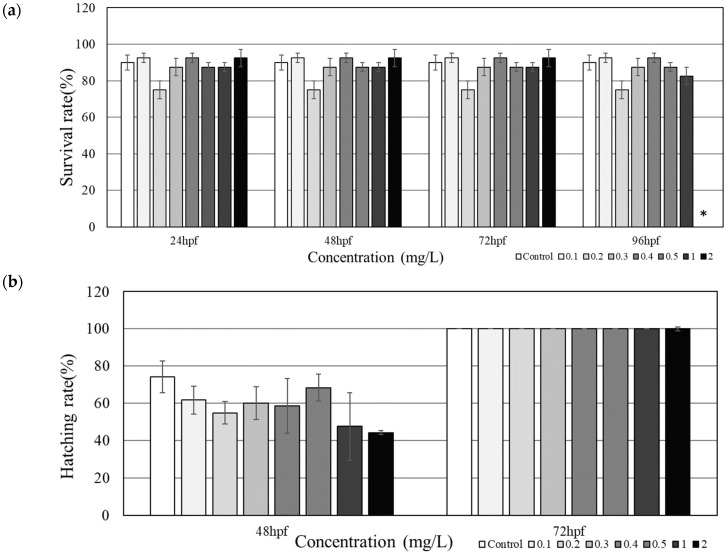
Survival (**a**) and hatching rate (**b**) of zebrafish embryos/larvae under polyhexamethylene guanidine-phosphate (PHMG-P) exposure (*N* = 10). Asterisks (*) denotes statistical significance (*p* < 0.05).

The treatment group under 2.0 mg/L PHMG-P showed 100% mortality after 96 h, while the rest of the groups did not show any significant changes (Figure 1a). Mortality was seen in all groups at 24 hpf, but no additional mortality was observed during 48 hpf to 72 hpf. An LC_25_ and an LC_50_ of tested PHMG-P in zebrafish was determined to be 0.4 mg/L and 1.2 mg/L, respectively.

**Figure 2:**
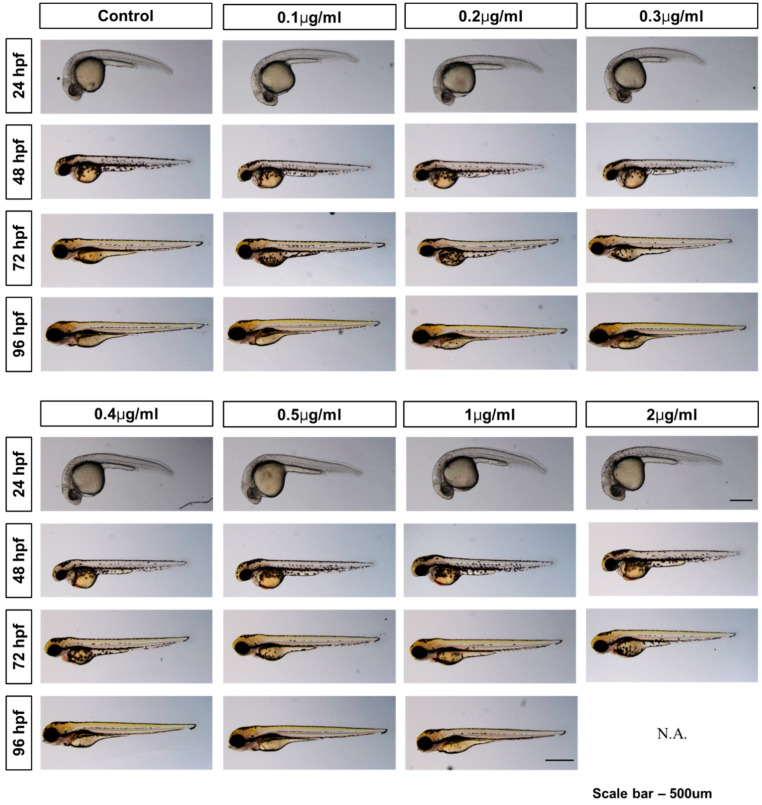
Morphological images of zebrafish embryos/larvae during embryogenesis under 0 to 2 mg/L PHMG-P exposure. N.A.: not available due to significant lethality.

At the 24 hpf timepoint, hatching was not seen for all embryos including the control and exposure groups. Hatching was observed at 48 hpf, with the control group having the highest hatching rate and the highest treatment group having the lowest, 74.2% and 44.2%, respectively (Figure 1b). All groups had successfully hatched by 72 hpf. Morphological images of zebrafish embryos/larvae were taken in groups of three to ensure accuracy in detecting for malformations (Figure S1). From 24 hpf to 96 hpf, no malformations parameters including bent spines, head malformation, and pericardial edema at the embryo/larval stage were observed (Figure 2).

### 3.2. Cardiotoxicity

**Figure 3:**
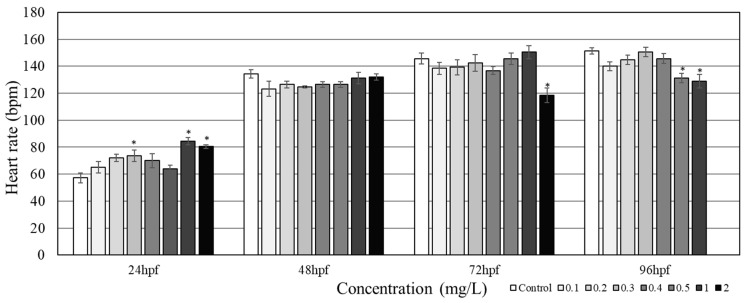
Heart rate of zebrafish embryos/larvae at 24 hpf, 48 hpf, 72 hpf, and 96 hpf under PHMG-P exposure (*N* = 5). Asterisk (*) denotes statistical significance *(p* < 0.05).

At 24 hpf, the heart rate for exposed groups steadily increased with increasing concentrations of PHMG-P up to 2.0 mg/L, except in groups exposed to 0.4 mg/L and 0.5 mg/L (Figure 3). Subsequently, significant increases in heart rate were observed in the zebrafish embryos under 0.3, 1.0, and 2.0 mg/L PHMG-P exposure. Interestingly, at 96 hpf, the heart rate of zebrafish larvae was reduced under 0.5 and 1.0 mg/L of PHMG-P exposure; the control group showed the highest average heart rate of 151.2 ± 2.3 bpm, and the treatment group under 1.0 mg/L of PHMG-P showed the lowest average of 128.8 ± 5.0 bpm.

### 3.3. RNA Sequencing Analysis

We found 76 GO enrichments significantly affected by PHMG-P exposure (0.4 mg/L) in the zebrafish larvae after 96 h exposure (Table 1). These enrichments were significant in all three individual replicate samples. The GO enrichments were related to biological processes (BP), immune response (GO:0002474, GO:0002376, GO:0006955, and GO:0045087) and inflammation (GO:0006954, GO:0030593, GO:0030595, and GO:0050900). Moreover, GO enrichment related to overall defense systems in zebrafish embryos/larvae were affected (GO:0002237, GO:0006950, GO:0006952, GO:0009607, GO:0009615, and GO:0009617).

### 3.4. Quantitative PCR Analysis

**Figure 4:**
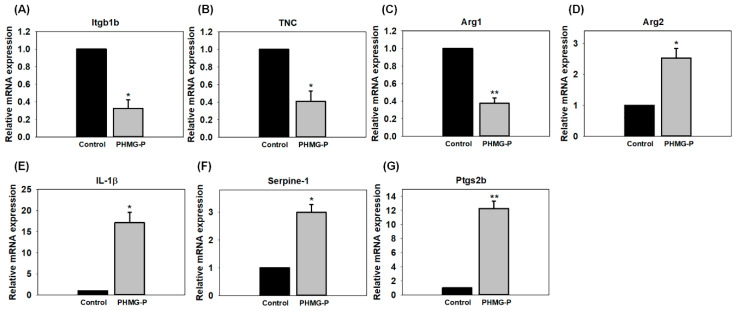
Gene transcription changes of *Itgb1b* (**A**), *TNC* (**B**), *Arg1* (**C**), *Arg2* (**D**), *IL-1β* (**E**), *Serpine-1* (**F**), and *Ptgs2b* (**G**) after 96 hpf PHMG-P exposure (*N* = 3). Asterisks (* and **) denotes statistical significance with *p* < 0.05 and *p* < 0.01, respectively.

To confirm the alteration of GO enrichment results, real-time PCR was performed. Seven immune response and inflammatory response-related genes *(integrin beta 1b2 (itgb1b), tenascin C (TNC), arginase1 (arg1), arginase2 (arg2), interleukin 1 beta (IL-1β), serpine-1, and prostaglandin endoperoxide synthase 2b (ptgs2b))* were significantly affected (Figure 4). The gene expressions of *Itgb1b*, *TNC*, and *Arg1* were significantly down-regulated in the zebrafish treated with 0.4 mg/L PHMG-P. However, those of *Arg2*, *IL-1β*, *Serpine-1*, and *Ptgs2b* were significantly upregulated.

## 4. Discussion

In the present study, we found out that, PHMG-P exposure suppresses hatching at high concentrations, and also causes mortality at high concentrations, even though morphological changes, including bent spines, head malformation and pericardial edema during embryogenesis were not observed. The zebrafish larvae exposed to 2 mg/L PHMG-P showed total mortality at 96 hpf (Figure 1a). The LC_50_ of PHMG-P for zebrafish embryos/larvae was derived at 1.2 mg/L (95% CI: 0.9661–1.3167 mg/L). A recent study reported 1.43 mg/L as the concentration showing an increase in coagulation for zebrafish embryos [12], matching well with the result of this present study. On the other hand, hatching was delayed at 48 hpf; however, all the zebrafish embryos were completely hatched without any significant differences at 72 hpf. (Figure 1b). At 48 hpf, the PHMG-P-exposed groups showed a lower hatching rate than the control group, the 1.0 and 2.0 mg/L groups showed a much lower hatching rate of 47.6% and 44.2%, respectively, though the difference was not statistically significant. This delayed hatching effect was also observed in the previous study [12]. This might suggest that PHMG-P suppresses the hatching mechanism. In a previous study, De la Paz et al. [23] found out that triazoles fungicides can block the secretory function of hatchling gland cells, by reduction of the release of choriolytic enzymes by the hatching gland cells. In our study, however, no morphological changes such as bent spines, head malformation, and pericardial edema were observed, although PHMG-P caused lethal and hatching delay.

Significant changes were seen in the zebrafish heart rates during the PHMG-P exposure period. The irregularities recorded might indicate cardiac dysfunction. One of the sensitive areas in zebrafish early development is the cardiovascular system [24], which is an easy target for dioxin-like toxins and pesticides, as these chemicals can weaken cardiovascular endothelial capacity by enacting oxidative stress signaling pathways. In our study, after 24 h, significant changes were observed in the heart rates of the 1.0 and 2.0 mg/L groups (Figure 3), which were higher than those in the control group. Interestingly, at 72 hpf, the 2.0 mg/L PHMG-P exposed group recorded the lowest heart rate and, consequently, a total mortality at 96 hpf. The sharp rise in heart rate for the first 24 h in both the 1.0 and the 2.0 mg/L PHMG-P exposed groups could be a result of a protective mechanism against the effects of the chemical exposure to the embryos. Previous studies show that some genes such as *Abcb4* in zebrafish serve as an active barrier against uptake of chemicals dissolved in water. *Abcb4* transcripts are constitutively expressed during the first 48 hrs of zebrafish embryo development [25]. Again, the notable increase in average heart rate of lower concentrations at 24 h may be an instance of hormesis. Similar readings were present in DMSO-treated embryos at lower concentrations [26]. Thus, the increasing heart rate of the 2.0 mg/L group, and the subsequent fall after 72 h and imminent death at 96 hpf, could have resulted from overspent energy during the formative stage in which additional transcriptome changes were observed (Table 1). This might suggest that PHMG-P could have some additional effects on zebrafish development, as studies show that some coplanar polychlorinated biphenyls affect formative heart cells [27].

PHMG-P exposure in rats and mice induced pulmonary inflammation and fibrosis [9,10]. In a coculture of bronchial epithelial cells, macrophages, and mast cells or human alveolar epithelial A549 cells, PHMG-P is reported to increase the production of proinflammatory cytokines and reactive oxygen species, and is also associated with the permeability of airway barriers [15,28]. In addition, Kim et al. [8] found that treatment of zebrafish with 0.3% PHMG-P resulted in attenuated development of zebrafish embryos and high serum triglyceride, fatty liver, and fibrous collagen levels in the bulbous artery of adult zebrafish. The genotypic changes reported in previous studies [9,10] were reproduced in this study by observing transcriptome changes. In the present study, genes related to immune response, inflammation, and defense systems were also significantly affected, though the results were derived from the whole body and not from a specific target organ. Hence, we found significant inflammatory damage to the zebrafish as a result of PHMG-P exposure from the RNA sequencing technique with GO analysis because RNA sequencing provides a transcriptome profile of zebrafish embryos where other splicing structures and uncommon transcripts can be identified [29].

Immune response and inflammatory related genes were significantly affected and were the predominate findings. Exposure of zebrafish embryo/larvae to PHMG-P is capable of causing inflammatory damage and deteriorates the immune response of the organism. In our study, transcriptome changes were confirmed by qPCR analysis (Figure 4). Levels of *Itgb1b* and *TNC* were significantly decreased. The reduced levels of cell adhesion or focal adhesion molecules such as *Itgb1b* and *TNC* may suggest a retarded development pathway [30]. *Itgb1b* and *TNC* are expressed during the embryonic developmental stages and play a critical role in immune responses and the activation of many intracellular signals. Specifically, *Itgb1b* and *TNC* are involved in growth and reformation. The latter enhances axonal regrowth and reformation after an injury, while the former grows the brachial arch [30,31]. These cell adhesion molecules are involved in binding with the extracellular matrix, where they maintain tissue integrity [32]. The movement of leukocytes from the bloodstream towards inflammatory foci is arbitrated by cell adhesion molecules [33]. Arginase also plays an important role in immune responses. Macrophage Arg1 is known to be involved in the resolution of inflammation and tissue repair by promoting T_h_2 cytokine production, which promotes cell proliferation and collagen production. In contrast, *Arg2* is associated with inflammatory responses [34,35,36]. Our results showed that *Arg1* and *Arg2* gene expression levels were 2.78-fold lower and 3.61-fold higher than those in the control group, respectively. Kim et al. reported that *Arg1* and *Arg2* are highly induced in PHMG-P-treated rats [11]. This difference in expression might arise from the sources of RNA. In the rat experiment, RNA was extracted from lung tissue that had been exposed to PHMG-P for four weeks [11], whereas in our zebrafish experiment, RNA was extracted from the whole body of zebrafish larvae. Arginase converts L-arginine into L-ornithine and urea and has two isoenzymes. *Arg1* is a cytosolic enzyme that functions in ammonia detoxification via involvement in the urea cycle in the liver, whereas *Arg2* is located in mitochondria and is expressed in extrahepatic tissues and cells such as the kidney, brain, small intestine, mammary gland, and macrophages [34]. L-arginine is metabolized into nitric oxide and L-citrulline by nitric oxide synthase. Arginase competes with nitric oxide synthase for their common substrate, L-arginine. In the lungs, bronchial epithelial cells, endothelial cells, (myo) fibroblasts, and alveolar macrophages express *Arg1* and *Arg2* [37,38]. The induction of arginase can downregulate nitric oxide production, which leads to the airway hyper responsiveness of chronic obstructive pulmonary disease, because nitric oxide functions in bronchodilation [39]. With zebrafish increasingly emerging as a substitute for rodent studies [28], we can establish based on rodent studies and this study that PHMG-P induces similar adverse effects as seen in rodent models.

Zebrafish preserves its role as a potent inflammatory cytokine involved in acute inflammatory responses, thus understanding the mechanism of inflammation in zebrafish during inflammatory response may shed light on its function in higher vertebrates including humans and mice [40]. Hence, it can be concluded that the exposure of PHMG-P to zebrafish embryos/larvae can cause inflammatory damage and may be directly related to pulmonary illness, since it has been associated with primary and secondary forms of pulmonary hypertension [39]. *IL-1β* is a strong pro-inflammatory cytokine that is critical for defense responses to infection and injury [41]. Interestingly, *IL-1β* showed a 17.1-fold increase compared to the control. Slightly elevated levels of *IL-1β* can induce the release of the adrenocorticotropic hormone, which produces leukocytosis and thrombocytosis [42]. Studies show that mice and rats treated with *IL-1β* showed a chronic fibrotic response in their lungs. In particular, *IL-1β* plays a direct role in acute and chronic inflammation and pulmonary fibrosis [43,44]. *Serpine1* serves as a mediator for epithelial cells and inflammatory activity. This gene’s transcription was increased by 2.99-fold in our study. In a recent study, Kaiko et al. reported that *Serpine1* was increased 6-fold in mice with colonic injury and inflammation [45]. *Serpine1* is upregulated in several cell types during injury, and it controls coagulation, fibrinolysis, and inflammation. Excessive release of *Serpine1* is associated with thrombophilia and inflammation-related injuries [15,46]. *Ptgs2b* gene transcription was also significantly increased by 12.27-fold. This signal might respond to injury or infection. Our findings appear to be well substantiated by studies on mRNA levels of zebrafish with wounded tail fins, i.e., based on the observations of qPCR analysis and significantly increased levels of the inflammation control marker, *ptgs2b* [47].

Due to different exposure routes between zebrafish and humans, the exposure levels of our study might not have environmental relevancy for humans. The aim of this study was also to identify the effects of PHMG-P. Nevertheless, it was important to compare the exposure concentrations of our study with the ones which humans were exposed to. Following the previous studies, PHMG-P concentrations in the two most popular humidifier disinfectant brands Oxy^®^ and Wiselect, were 1276 mg/L and 1307 mg/L, respectively [48,49]. Considering a dilution ratio of 200:1 from the products to airborne, the diluted concentration of PHMG-P in the air was approximately 6.5 mg/L. Part of our exposure levels were in an order level, although a direct comparison was inappropriate.

In this study, the toxicity of PHMG-P to zebrafish embryos/larvae resulting from transcriptome changes was assessed by RNA sequencing and qPCR confirmation. Furthermore, we evaluated phenotypic changes in zebrafish embryos/larvae, such as hatchability, survival, malformation, or heart rate. Certain common effects on immune and inflammatory systems were found using transcription analysis.

## Figures and Tables

**Table 1:** Significantly affected gene ontologies (GOs) after PHMG-P exposure to zebrafish embryo/larvae.

Ontology	Term	GO ID	*p*-Value (at 96 h)		
			I	II	III
**BP**	response to molecule of bacterial origin	GO:0002237	0.000006	0.000002	0.000388
**BP**	immune system process	GO:0002376	<0.000001	<0.000001	<0.000001
**BP**	antigen processing and presentation of peptide antigen via MHC class I	GO:0002474	0.000917	0.000005	0.000082
**BP**	UDP-N-acetylglucosamine metabolic process	GO:0006047	0.000256	0.000352	0.000321
**BP**	protein glycosylation	GO:0006486	0.000252	0.000006	0.000049
**BP**	proteolysis	GO:0006508	0.000184	0.000172	0.000098
**BP**	fatty acid biosynthetic process	GO:0006633	<0.000001	<0.000001	0.000011
**BP**	unsaturated fatty acid biosynthetic process	GO:0006636	0.000002	0.000003	0.000035
**BP**	response to stress	GO:0006950	<0.000001	<0.000001	<0.000001
**BP**	defense response	GO:0006952	<0.000001	<0.000001	<0.000001
**BP**	inflammatory response	GO:0006954	<0.000001	<0.000001	<0.000001
**BP**	immune response	GO:0006955	<0.000001	<0.000001	<0.000001
**BP**	nucleotide-sugar biosynthetic process	GO:0009226	0.000001	0.000001	0.000001
**BP**	GDP-mannose biosynthetic process	GO:0009298	0.000350	0.000448	0.000418
**BP**	response to biotic stimulus	GO:0009607	<0.000001	<0.000001	<0.000001
**BP**	response to wounding	GO:0009611	<0.000001	<0.000001	<0.000001
**BP**	response to virus	GO:0009615	<0.000001	<0.000001	<0.000001
**BP**	response to bacterium	GO:0009617	<0.000001	<0.000001	<0.000001
**BP**	cytokine-mediated signaling pathway	GO:0019221	0.000032	0.000068	0.000238
**BP**	GDP-mannose metabolic process	GO:0019673	0.000018	0.000025	0.000023
**BP**	neutrophil chemotaxis	GO:0030593	0.000323	0.000471	0.000049
**BP**	leukocyte chemotaxis	GO:0030595	0.000010	0.000018	0.000002
**BP**	response to lipopolysaccharide	GO:0032496	0.000022	0.000006	0.000216
**BP**	response to cytokine	GO:0034097	0.000006	0.000015	0.000011
**BP**	positive regulation of MAPK cascade	GO:0043410	0.000206	0.000363	0.000002
**BP**	innate immune response	GO:0045087	0.000051	0.000008	<0.000001
**BP**	leukocyte migration	GO:0050900	<0.000001	0.000002	<0.000001
**BP**	cell chemotaxis	GO:0060326	0.000016	0.000029	0.000003
**CC**	cell-cell junction	GO:0005911	0.000003	0.000011	0.000345
**CC**	tight junction	GO:0005923	<0.000001	<0.000001	0.000026
**CC**	NADPH oxidase complex	GO:0043020	0.000133	0.000172	0.00016
**CC**	apical junction complex	GO:0043296	0.000001	<0.000001	0.000042
**MF**	alpha-1,6-mannosyltransferase activity	GO:0000009	0.000383	0.000125	0.000105
**MF**	alpha-1,2-mannosyltransferase activity	GO:0000026	0.000438	0.000146	0.000123
**MF**	alpha-1,3-mannosyltransferase activity	GO:0000033	0.000383	0.000125	0.000105
**MF**	alpha-1,3-galactosyltransferase activity	GO:0001962	0.000383	0.000125	0.000105
**MF**	NAD+ ADP-ribosyltransferase activity	GO:0003950	<0.000001	0.000354	0.000034
**MF**	UDP-glucose:glycoprotein glucosyltransferase activity	GO:0003980	0.000501	0.000170	0.000143
**MF**	endopeptidase activity	GO:0004175	<0.000001	<0.000001	<0.000001
**MF**	cysteine-type endopeptidase activity	GO:0004197	<0.000001	<0.000001	<0.000001
**MF**	glycolipid mannosyltransferase activity	GO:0004376	0.000498	0.000169	0.000142
**MF**	oligosaccharyl transferase activity	GO:0004576	0.000693	0.000247	0.000208
**MF**	dolichyl-phosphate-glucose-glycolipid alpha-glucosyltransferase activity	GO:0004583	0.000437	0.000146	0.000122
**MF**	phospholipase inhibitor activity	GO:0004859	0.000001	0.000002	0.000001
**MF**	peptidase activity	GO:0008233	0.000010	0.000030	0.000003
**MF**	cysteine-type peptidase activity	GO:0008234	0.000038	0.000103	<0.000001
**MF**	acetylglucosaminyltransferase activity	GO:0008375	0.000624	0.000070	0.000056
**MF**	galactosyltransferase activity	GO:0008378	0.000194	0.000002	0.00001
**MF**	fucosyltransferase activity	GO:0008417	0.000003	0.000001	0.000006
**MF**	O antigen polymerase activity	GO:0008755	0.000383	0.000125	0.000105
**MF**	lipopolysaccharide-1,6-galactosyltransferase activity	GO:0008921	0.000383	0.000125	0.000105
**MF**	transferase activity, transferring glycosyl groups	GO:0016757	0.000001	<0.000001	0.000001
**MF**	transferase activity, transferring hexosyl groups	GO:0016758	0.000320	0.000001	0.000027
**MF**	cellulose synthase activity	GO:0016759	0.000383	0.000125	0.000105
**MF**	9-phenanthrol UDP-glucuronosyltransferase activity	GO:0018715	0.000383	0.000125	0.000105
**MF**	1-phenanthrol glycosyltransferase activity	GO:0018716	0.000383	0.000125	0.000105
**MF**	9-phenanthrol glycosyltransferase activity	GO:0018717	0.000383	0.000125	0.000105
**MF**	1,2-dihydroxy-phenanthrene glycosyltransferase activity	GO:0018718	0.000383	0.000125	0.000105
**MF**	phenanthrol glycosyltransferase activity	GO:0019112	0.000383	0.000125	0.000105
**MF**	beta-1,4-mannosyltransferase activity	GO:0019187	0.000383	0.000125	0.000105
**MF**	alpha-1,2-galactosyltransferase activity	GO:0031278	0.000383	0.000125	0.000105
**MF**	dolichyl pyrophosphate Man7GlcNAc2 alpha-1,3-glucosyltransferase activity	GO:0033556	0.000383	0.000125	0.000105
**MF**	UDP-glucosyltransferase activity	GO:0035251	0.000810	0.000294	0.000248
**MF**	lipopolysaccharide-1,5-galactosyltransferase activity	GO:0035496	0.000383	0.000125	0.000105
**MF**	dolichyl pyrophosphate Man9GlcNAc2 alpha-1,3-glucosyltransferase activity	GO:0042281	0.000497	0.000169	0.000142
**MF**	dolichyl pyrophosphate Glc1Man9GlcNAc2 alpha-1,3-glucosyltransferase activity	GO:0042283	0.000383	0.000125	0.000105
**MF**	inositol phosphoceramide synthase activity	GO:0045140	0.000383	0.000125	0.000105
**MF**	alpha-(1->3)-fucosyltransferase activity	GO:0046920	0.000059	0.000022	0.000099
**MF**	alpha-(1->6)-fucosyltransferase activity	GO:0046921	0.000438	0.000146	0.000123
**MF**	indole-3-butyrate beta-glucosyltransferase activity	GO:0052638	0.000383	0.000125	0.000105
**MF**	salicylic acid glucosyltransferase (ester-forming) activity	GO:0052639	0.000383	0.000125	0.000105
**MF**	salicylic acid glucosyltransferase (glucoside-forming) activity	GO:0052640	0.000383	0.000125	0.000105
**MF**	benzoic acid glucosyltransferase activity	GO:0052641	0.000383	0.000125	0.000105
**MF**	chondroitin hydrolase activity	GO:0052757	0.000383	0.000125	0.000105
**MF**	dolichyl-pyrophosphate Man7GlcNAc2 alpha-1,6-mannosyltransferase activity	GO:0052824	0.000383	0.000125	0.000105
**MF**	cytokinin 9-beta-glucosyltransferase activity	GO:0080062	0.000383	0.000125	0.000105

MF: Molecular Function, CC: Cellular Components, BP: Biological Process. GO terms with *p* < 0.05 were listed in this table. I, II, III indicates each replicate.

## Supplementary Materials

The following are available online at https://www.mdpi.com/2305-6304/8/2/33/s1, Figure S1: Morphological images as groups during embryogenesis under 0 to 2 mg/L PHMG-P exposure, Table S1: List of primers for real-time PCR.
