# Supplementary material for: A Phenotypic and Genotypic Evaluation of Developmental Toxicity of Polyhexamethylene Guanidine Phosphate Using Zebrafish Embryo/Larvae
Source: Toxics. 2020 May 2;8(2):33. doi: 10.3390/toxics8020033 (PMC7355787; doi:10.3390/toxics8020033)
Supplement: Supplementary file 1 [file toxics-08-00033-s001.pdf]

Supplementary Materials: A Phenotypic and Genotypic Evaluation of Developmental Toxicity of Polyhexamethylene Guanidine Phosphate Using Zebrafish Embryo/Larvae

Jeongah Song, Kojo Eghan, Sangwoo Lee, Jong-Su Park, Seokjoo Yoon, Wittaya Pimtong and Woo-Keun Kim

Table S1. List of primers for real-time PCR.

Gene	GenBank ID	Forward Primer	Reverse Primer	Amplicon
integrin, beta 1b.2 (itgb1b.2)-1	NM_212928.2	TCGATGTATGTGCGGGC AGT	CCACATTCACAGATGC CGTGT	132
tenascin C (TNC)	NM_001312316.1	CGTGACGTGACGGACA CAAC	ACAGAGATGCCGTCC ACCTG	71
Arginase 1 (Arg1)	NM_001045197	GGTGTGCAGGAAGGAG CAGA	CGATGGACTCGTCGTT GGGA	127
Arginase 2 (Arg2)	NM_199611	AGGAAGCAAAGGCCGA TCCA	CTGTAGGTCAGTCCGC CGTT	101
IL-1β	NM_212844.2	TGGCGAACGTCATCCAA G	GGA GCA CTG GGC GAC GCA TA	220
serpine 1	NM_001114559.1	TTGCTGAAGCCGTCCAG TCT	CCATCTTGGAGGCGAG CAGT	129
prostaglandinend operoxide synthase 2b (ptgs2b)	NM_001025504.2	GCTGACATCACGTGCCC ATC	TACCTGCGACTCCCAT TGGC	155
ribosomal protein P0 (rpp0)	NM_131580	CTGAACATCTCGCCCTT CTC	TAGCCGATCTGCAGAC ACAC	161

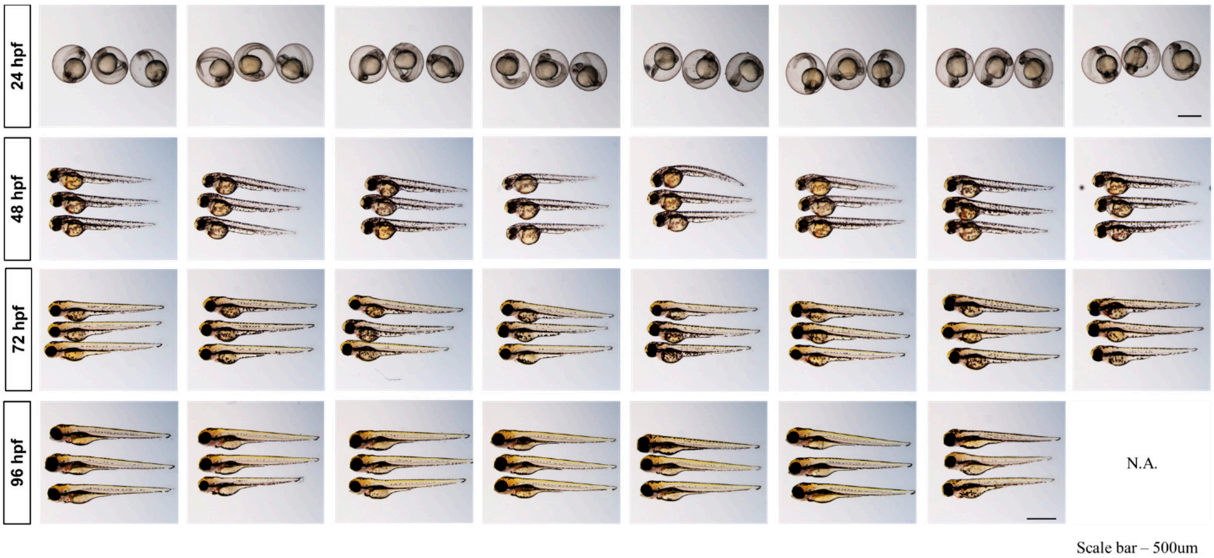

Figure S1. Morphological images as groups during embryogenesis under 0 to 2 mg/L PHMG-P exposure. N.A.: not available due to significant lethality.
